# Developing a viva exam to assess clinical reasoning in pre-registration osteopathy students

**DOI:** 10.1186/1472-6920-14-193

**Published:** 2014-09-19

**Authors:** Paul Orrock, Sandra Grace, Brett Vaughan, Rosanne Coutts

**Affiliations:** School of Health & Human Sciences, Southern Cross University, Lismore, Australia; College of Health & Biomedicine, Victoria University, Melbourne, Australia; Institute of Sport, Exercise & Active Living, Victoria University, Melbourne, Australia

## Abstract

**Background:**

Clinical reasoning (CR) is a core capability for health practitioners. Assessing CR requires a suite of tools to encompass a wide scope of contexts and cognitive abilities. The aim of this project was to develop an oral examination and grading rubric for the assessment of CR in osteopathy, trial it with senior students in three accredited university programs in Australia and New Zealand, and to evaluate its content and face validity.

**Methods:**

Experienced osteopathic academics developed 20 cases and a grading rubric. Thirty senior students were recruited, 10 from each university. Twelve fourth year and 18 fifth year students participated. Three members of the research team were trained and examined students at an institution different from their own. Two cases were presented to each student participant in a series of vignettes. The rubric was constructed to follow a set of examiner questions that related to each attribute of CR. Data were analysed to explore differences in examiner marking, as well as relationships between cases, institutions, and different year levels. A non-examining member of the research team acted as an observer at each location.

**Results:**

No statistical difference was found between the total and single question scores, nor for the total scores between examiners. Significant differences were found between 4^th^ and 5^th^ students on total score and a number of single questions. The rubric was found to be internally consistent.

**Conclusions:**

A viva examination of clinical reasoning, trialled with senior osteopathy students, showed face and content validity. Results suggested that the viva exam may also differentiate between 4^th^ and 5^th^ year students’ capabilities in CR. Further work is required to establish the reliability of assessment, to further refine the rubric, and to train examiners before it is implemented as a high-stakes assessment in accredited osteopathy programs.

**Electronic supplementary material:**

The online version of this article (doi:10.1186/1472-6920-14-193) contains supplementary material, which is available to authorized users.

## Background

Clinical reasoning (CR) is a core capability for health practitioners to problem solve, both in pre-entry level training and ongoing clinical practice. It has been described as “… *a context*-*dependent way of thinking and decision making on professional practice to guide practice actions*” (p.4) [1]. CR involves a series of cognitive processes where a practitioner gathers information about a patient, synthesises that information then develops and implements a treatment and management strategy. Reasoning also goes beyond the cognitive to the meta-cognitive and takes into account the practitioner’s capacity for self-appraisal and self-monitoring. Simmons [2] lists the attributes of the CR process as analysis, deliberation, heuristics, inference, metacognition, logic, cognition, information processing and intuition, which blend together and are weighted differently according to the practice setting, level of the practitioner’s experience, life experience, maturity and cognitive ability [2]. Dual process theory highlights the interaction between two fundamental approaches to clinical reasoning: non-analytic (intuitive, pattern recognition) reasoning (System 1) and analytical reasoning (hypothetico-deductive) reasoning (System 2) [3]. There is debate about whether the integration of these diverse processes develops only with experience or can be taught and learnt during training [4–7]. Research in physiotherapy has also highlighted that practitioners move between reasoning about patients physical complaints using analytic or non-analytic reasoning and engaging with the patient or their carer (narrative reasoning) to understand the impact of the presenting complaint [8].

### Clinical reasoning in osteopathic practice

Osteopathy is a primary care, limited-scope practice in most countries outside the USA, where it is a full scope medical practice. CR is a core skill in the practice of osteopathy and training institutions in Australia are required to demonstrate a coherent pedagogy for teaching and assessing this skill in order to be accredited to graduate registerable practitioners. Knowledge application, knowledge generation, problem-solving, analysis, and justification of clinical decisions/judgment are central among the requirements for developing osteopathic clinical skills in the pre-professional curricula [9]. CR in osteopathy has only recently been discussed in the literature [10–12]. It has been suggested that reasoning for experienced osteopaths lies along a continuum from technical rationality, encompassing a practitioner-centred, biomedical and biomechanical approach, to professional artistry that is a more patient-centred holistic approach [13].

### Teaching clinical reasoning

Developing such a complex capability as clinical reasoning requires careful scaffolding throughout the curriculum. Each of the key elements of CR in case management, including data acquisition, diagnostic development, and dual processing of information need to be developed and assessed in turn and later as an integrated process [14]. The parallel skill of critical thinking is commonly developed in science subjects and gradually integrated with case-based learning as students progress through their programs of study [15]. Cases also require increasingly complex problem solving, informed by their stage of learning (novice, advanced beginner, competent and proficient practitioner and expert) [16]. Problem-based learning (PBL) has been reported to enhance student achievement in a number of skills, including the application of problem solving in new situations, the ability to apply creative and critical thought, and the adoption of a holistic approach to problems and situations [17–20]. PBL has also been proposed to improve the integration of critical thinking in the osteopathic approach to CR [21].

It has been reported that repeated exposure to real cases early in the curriculum is beneficial, particularly when students are coached by skilled clinical teachers [5] who understand the importance of dual processing [22]. This ensures that errors in their thought processes are pointed out and students are given time to reflect in real time. The training of clinical teachers is pivotal to ensure recognition of differences between the way experts reason compared with students, and to clarify expectations of clinical reasoning abilities in students of different levels [23].

Recent research has emphasised the collaborative and ethical aspects of clinical reasoning [24–26]. However, attention to developing such aspects of clinical reasoning may be limited or absent from curricula. In one study of a professional entry physiotherapy course, key clinical reasoning dimensions like reflective practice and dialectical thinking were found to be under-developed [27].

### Assessing clinical reasoning

Assessing CR typically uses a simulated authentic clinical stimulus to elicit written, verbal or practical performance responses. It may also be assessed on an informal basis such as during clinical teaching rounds [18, 28]. CR tests like the Clinical Reasoning Problem or the Clinical Reasoning Exercise [29] have been developed. However, our current understanding of CR suggests that problem solving is largely dependent on the amount, specificity and organisation of knowledge possessed by the student. There is apparently no evidence among experts for the existence of a general problem-solving skill that is independent of content [30]. It also appears that simulation technologies with capacity for much greater sampling are more valid instruments than complex clinical simulations [30]. Current strategies for assessing clinical reasoning based on real life case studies include [19, 31, 32]:

- Key Features Question [33–35] - key features are those essential for resolving a problem. The format allows for examination of a large number of cases in a short timeframe;
- Vignette-based multiple choice questions - short vignettes are presented and responses to judgements or decisions about any aspect of the case are called for from a list of options however these only assess the *technical rational* aspect of reasoning [36];
- Extended matching questions [37–39] - students are required to match a series of brief case scenarios based on a single complaint with the most appropriate diagnosis or action;
- Problem based scenarios that present aspects of a case in steps, each one requiring recording of student reasoning and investigation of knowledge sources for solutions to queries; and
- Script Concordance Tests (SCT) [40] - use case based scenarios with a series of questions prompting further diagnostic thought and analysis, conducted online or in person.

However, such assessments on their own are likely to focus narrowly on discipline-specific cognitive skills. These assessments focus on diagnostic reasoning only and are unlikely to demonstrate the more global development of students’ attitudes, and particularly of their ability to reflect on and critique ways of both knowing and acting in the world beyond the clinic. The process of reasoning in diagnosis is predominantly deductive, especially in acute presentations where there is a need for medical intervention. However, it is only part of practice and does not cover the subsequent implications of management and prognostication. Reflective journals, oral case discussions, participation in collaborative practices, observation of clinical practice or parts of consultations (e.g. Objective Structured Clinical Examinations, timed station-based examinations that can include case vignettes requiring an oral or written reasoned decision processes) [41], and viva voce exams may more fully contribute to developing critical practitioners than assessments focussed narrowly on cognitive skills. The use of an oral exam based on simulated cases has been reported to be a reliable [42] and valid [43] method for assessing clinical reasoning when the case scenarios and marking criteria are consistently applied. While there is no reliable gold standard for assessing such a complex skill, a suite of methods to evaluate one or more aspects of the clinical reasoning process appears to be the most evidence-based approach [7]. A grading rubric is an assessment tool that describes the expectations and performance criteria of an exam explicitly [44], thereby enhancing examiner marking reliability [45]. The use of a grading rubric that is developed collaboratively and is authentic to clinical examiners language and understanding establishes its validity for the assessment task [45].

CR is assessed in pre-registration high and low stakes clinical examinations in Australian osteopathic courses. Moore et al. [46] reported on a project to benchmark the assessment of clinical reasoning in osteopathic curricula in Australia, New Zealand and the UK. These authors found that a range of tools are used to assess clinical reasoning including OSCEs, supervisor’s reports and oral exams. According to Vaughan et al. [47], universities teaching osteopathy programs commonly include an oral (viva voce) assessment with a number of variations. There has been no published research that analyses the validity or reliability of this assessment approach in osteopathic education.

The aim of this research was to construct an oral case exam that specifically tests clinical reasoning in osteopathy, to trial this exam with senior osteopathic students in the participating institutions, and to evaluate its content and face validity.

## Methods

This study was approved by the Human Research Ethics Committee’s of Southern Cross University, Unitec and Victoria University.

### Designing the assessment

#### Marking sheet

The research team met to develop the content of the assessment sheet around the attributes associated with CR proposed by Simmons for nursing students. Criteria outlined by Simmons [2] were considered by the research group to have synergies with the reasoning process in osteopathy; moreover the language that was used to describe the criteria was readily understandable to potential examiners. For the purposes of the assessment, the headings from Simmons were called ‘attributes’. One or more questions for each attribute were developed for examiners to ask students during the assessment. The research team developed a marking rubric that incorporated a 1-5 scale, with each level described (Additional file 1). The rubric performance criteria were based on the established learning objectives of 4^th^ and 5^th^ year clinical students in a previous benchmarking exercise [46]. The marking standards for each attribute were established both during the case development and also reflecting the previous experience of examiner teams using these cases for clinical exams.

#### Cases

Twenty cases were developed for the assessment. Cases 1-10 were termed ‘short cases’ and contained brief information about the case. Cases 11-20 were termed ‘long cases’ and contained more detailed information about the case and the patient. Examples of each case type are found at Additional file 2. The cases used were chosen from banks of cases that had been developed by osteopathic staff at both SCU and VU. At SCU the content validity of cases was confirmed in a series of focus groups of experienced clinicians; at VU via a reference group from multiple clinics and universities. The cases used in this study were chosen by the research team based on their authenticity, complexity and presence of cues to other systems and possibilities. Simple acute or uncomplicated red flag cases were avoided.

### Participants

#### Students

Thirty (n = 30) students from the participating institutions responded to an invitation to participate in the study, and ten (n = 10) students from each institution were available during the assessment period. All students were either enrolled in year 4 or year 5 of the teaching programs. At the time of the study (October-November 2013), the year 4 students were moving into their final year of the program, and the year 5 students were graduating. The students were informed that the examination would have no bearing on any of their results or progression through the teaching program, and that the primary aim of the study was to develop the assessment tool. The cases used in this study were new to the students who were examined. There were example cases given to students so they understood the examination process. Participating students were able to receive immediate feedback from the examiner about their performance for their own learning.

#### Examiners

Three examiners were recruited, one from each of the three participating universities. Each examiner examined students enrolled in an institution other than their own. Before the examination, the examiners met by Skype (Skype Inc.) to discuss each case and develop a consensus as to the expected performance levels for each of the criteria on the assessment rubric. All three examiners were registered osteopaths with over 10 years experience in clinical assessments and assessment design. Each examiner also acted as an observer when the examination was conducted at their own institution. This process was employed to ensure consistency and fairness and to provide feedback about the assessment as part of a quality improvement process.

### Assessment process

The assessment for each student ran for 30 minutes. During that time each student was presented with one ‘short case’ and one ‘long case’ that were unique for each student. The student had 15 minutes to answer the questions for each case from the examiner. All questions from the examiner were taken from the assessment rubric and the examiner was required to only ask those questions and ask them in order. The information from each case was divided into three parts; initial history, further information, and examination findings. Students were provided with the first part and then asked to discuss their thoughts on the case based on the examiner questions. The student was then presented with the second part and further discussion took place based on the examiner’s questions. This was repeated for the third part of the case. The examiner marked the first case prior to proceeding to the next case.

### Data analysis

Data from each of the assessment sheets were entered into Microsoft Excel by a research assistant and then transposed to SPSS Version 21 (IBM Corp, USA) for analysis. Descriptive statistics were generated for each case and each question on the assessment rubric. An ANOVA was used to assess for statistically significant differences between the total scores for each case along with differences between the total scores and question scores awarded by each examiner. Alpha was set at p < 0.05. Cronbach’s alpha was used to assess the internal consistency of the assessment rubric and Pearson’s *r* was used to correlate each question on the assessment rubric. The total and question scores from the students’ first and second cases were compared to examine whether their performance on the first case predicted their performance on the second case [28]. Pearson’s *r* was interpreted according to Hopkins [48]: <0.10 (trivial); 0.10-0.30 (small); 0.30-0.50 (moderate); 0.50-0.70 (large); 0.70-0.90 (very large); 0.90-1.0 (perfect). Partial eta-squared (η^2^_p_) was reported for the total score between the year levels.

## Results

Descriptive statistics for each question on the assessment rubric are presented in Table 1, and for each year level and university in Table 2.

**Table 1:** **Descriptive statistics for each question and total score on the assessment rubric**

	Range	Minimum	Maximum	Mean	Std. Deviation
**Q1**	3	2	5	3.58	.74
**Q2**	3	2	5	3.52	.65
**Q3**	3	2	5	3.67	.75
**Q4**	3	2	5	3.65	.78
**Q5**	3	2	5	3.42	.59
**Q6**	3	2	5	3.05	.72
**Q7**	3	2	5	3.12	.90
**Q8**	3	2	5	3.33	.70
**Q9**	3	2	5	3.32	.68
**Q10**	3	2	5	3.42	.74
**Q11**	3	2	5	3.32	.85
**Q12**	3	2	5	3.07	.82
**Q13**	2	2	4	2.88	.58
**Q14**	3	1	4	2.97	.76
**Total**	30	31	61	46.23	7.82

**Table 2:** **Descriptive statistics for each year level**, **university**

	Total score	N	Mean	Std. deviation	Std. error mean
**Year level**	Year 4	12	43.59	8.05	2.32
	Year 5	18	48.71	6.83	1.61
**University**	SCU	10	48.65	8.23	2.60
	Unitec	10	45.95	8.67	2.74
	VU	10	44.10	5.97	1.88

A number of questions were typically scored higher by the examiners for year 5 students when compared to their year 4 counterparts. These questions were Question 3. *What are the primary cues in the additional case information*?, Question 7. *Please summarise the case so far*; *including your thoughts on differentials*, *examination and treatment strategies*, 8. *Give your reasoning for choice of differentials*?, Question 11. *Can you tell me about alternative diagnostic or treatment choices if what you have planned doesn*’*t work*?, Question 13. *What are your thoughts about how your handling of this case could have been improved*?, Question 14. *How did the osteopathic principles influence your reasoning in this case*? and the total score.

### Between groups analysis

There were no statistically significant differences (p > 0.05) between the total and question scores for the individual cases, or between case types (short versus long case).

There were no statistically significant differences between examiners for the total score (F_(2,57)_ = 1.75, p = 0.182). Significant differences were revealed between examiners for Question 7 (F_(2,57)_ = 6.22, p = 0.004) and Question 12 (F_(2,57)_ = 4.51, p = 0.015). Post hoc testing showed the difference at Question 7 to be between Examiner 1 and Examiner 2 (p = 0.003), and Examiner 1 and Examiner 3 (p = 0.043). The mean score for Examiner 1 was lower (2.60 +/- 0.68) compared to both Examiner 2 (3.50 +/- 0.89) and Examiner 3 (3.25 +/- 0.91). For Question 12, there was a statistically significant difference between Examiner 1 and Examiner 2 (p = 0.016). The mean score for Examiner 1 for this question was lower (2.65 +/- 0.67) compared to Examiner 2 (3.35 +/- 0.81).

Significant differences were noted between student year levels for Question 3 (F_(1,58)_ = 5.06, p = 0.028), Question 6 (F_(1,58)_ = 5.06, p = 0.020), Question 7 (F_(1,58)_ = 10.18, p = 0.002), Question 8 (F_(1,58)_ = 4.57, p = 0.037), Question 11 (F_(1,58)_ = 13.98, p < 0.001), Question 13 (F_(1,58)_ = 6.75, p = 0.012), Question 14 (F_(1,58)_ = 6.25, p = 0.015) and the total score (F_(1,58)_ = 7.09, p = 0.010, η^2^_p_ = 0.109). The mean scores for these questions and the total score were higher in the year 5 students compared to students in year 4. The student’s year level accounted for approximately 11% of the variance in the total score.

### Internal consistency and correlations

The internal consistency of the assessment rubric was α = 0.944. The alpha score did not improve if any of the questions were removed. Large correlations (*r* >0.70) were observed for the question combinations highlighted in bold in Table 3.

**Table 3:** **Assessment rubric question correlations**

	Q1	Q2	Q3	Q4	Q5	Q6	Q7	Q8	Q9	Q10	Q11	Q12	Q13	Q14
**Q1**	1													
**Q2**	.**838** ^**^	1												
**Q3**	.657^**^	.670^**^	1											
**Q4**	.**712** ^**^	.632^**^	.**725** ^**^	1										
**Q5**	.518^**^	.489^**^	.547^**^	.618^**^	1									
**Q6**	.513^**^	.520^**^	.530^**^	.424^**^	.585^**^	1								
**Q7**	.604^**^	.530^**^	.532^**^	.590^**^	.669^**^	.639^**^	1							
**Q8**	.658^**^	.616^**^	.597^**^	.649^**^	.638^**^	.665^**^	.**736** ^**^	1						
**Q9**	.672^**^	.585^**^	.578^**^	.601^**^	.598^**^	.522^**^	.576^**^	.**770** ^**^	1					
**Q10**	.565^**^	.529^**^	.526^**^	.521^**^	.525^**^	.528^**^	.507^**^	.668^**^	.**711** ^**^	1				
**Q11**	.452^**^	.494^**^	.484^**^	.400^**^	.473^**^	.551^**^	.654^**^	.582^**^	.528^**^	.510^**^	1			
**Q12**	.435^**^	.442^**^	.586^**^	.515^**^	.571^**^	.537^**^	.629^**^	.635^**^	.603^**^	.454^**^	.**719** ^**^	1		
**Q13**	.432^**^	.295^*^	.527^**^	.393^**^	.388^**^	.415^**^	.443^**^	.548^**^	.609^**^	.543^**^	.483^**^	.581^**^	1	
**Q14**	.456^**^	.345^**^	.545^**^	.354^**^	.372^**^	.529^**^	.525^**^	.560^**^	.517^**^	.476^**^	.357^**^	.521^**^	.641^**^	1

**correlation is significant at p < 0.01. Large (>0.7) correlations are highlighted in bold.

The observer comments reflected that the process was generally consistent. There were reports of some variation in the consecutive process of the questions, which improved as each examiner became more experienced in the format. It was noted that the time limit of 15 minutes per case meant that examiners ran late if the student required prompting or clarification. Also noticed was a tendency to lead the student to certain answers with any clarifications and prompting. Further examiner training would ensure that the process was made more robust.

## Discussion

The aim of the present study was to develop, pilot and provide feedback following a viva examination of clinical reasoning in osteopathy. Students in the final two years of their five-year osteopathy program participated. The conduct of the examination developed in the present study was based the Script Concordance Test (SCT) [40]. The use of the SCT as the basis for the exam was appropriate, as it would allow students to ‘move’ through the case as they were presented with new information. The SCT has previously been described in the osteopathic education literature [49] although it is not widely used in the profession at present. An assessment rubric was developed based on the work of Simmons [2] for nursing students. This model was appropriate for osteopathic practice although the assessment questions presented by Roberts [50] were also considered. The Roberts rubric had similar heuristics to Simmons, but was developed to be used in real practice. Simmons’ rubric was based more specifically on concepts of CR and was better able to be adapted for use in a simulated oral case examination.

Face and content validity were established for the assessment rubric by presenting it to experienced osteopathic academics and clinical educators. Input from multiple ‘experts’ is important as experts differ in their expectations and processes for how a problem may be reasoned [23]. This process ensured that the rubric was inclusive of all inputs from experts about CR in osteopathic practice.

Cases that were reflective of the typical conditions that present to Australasian osteopathic practice [51, 52] were developed and tested as part of the current study. The cases contained information about the patient and their presenting complaint, medical history and psychosocial history, and were presented to students in three stages. At each stage the students were required to ‘think-aloud’ about their approach to reasoning the theoretical case and this is something that has not previously been published in osteopathy. The think-aloud approach has been used widely to teach [53, 54] and qualitatively investigate CR [55–57]. However, its use in summative assessments of CR specifically, has not been demonstrated. The assessment rubric (Additional file 1) contained a series of prompt questions for the examiner to ask the student at each stage. The examiners were required to ask the same questions of each student in order to standardise the examination. One of the examiners initially reported this to be quite difficult as they wanted to explore the students’ reasoning process beyond the standard questions however the observer reminded them of the examination process. Further examiner training will assist in ensuring that all examiners ask the standard questions.

Each question related to one of the attributes on the assessment rubric. These attributes focus on the decision making process and management of the patient [28]. The lowest mean score was for Question 14. *How did the osteopathic principles influence your reasoning in this case*? Given that this was an assessment of CR in osteopathy, it is interesting to observe that the only question explicitly containing the word ‘osteopathic’ was consistently rated lowest by the examiners. This is likely due to a combination of two factors: 1) the examiners applying their own ideas and expectations as to how the osteopathic principles should have influenced the students’ response; and 2) the students having difficulty explicating how the osteopathic principles influenced their reasoning. The highest mean score occurred for Question 3. *What are the primary cues in the additional case information*? Students taking part in this study were in the final two years of the programme when they see patients independently under supervision, and as such are required to present a rationale for the treatment and management plan for each patient as part of their clinical training. Students are likely to be comfortable discussing how they would manage a patient like the one described in the case even though they have not directly talked to or observed the patient.

There were no significant differences between the total or question scores for each of the 20 cases. This information provides an indication as to the comparability of students performances on each of the cases, and that one case may not be significantly harder or easier than another, despite the fact that there were both short and long cases with different depths of information provided. Real cases will vary in their complexity, and experts could be asked to rate the complexity of each case to ensure that examiners assess students fairly across the cases.

The results suggest that there is no significant difference between the scores awarded based on case type (short or long case). From a practical standpoint, the feedback obtained from the observers at all three institutions highlighted that there may be too much information in the long cases which increases the length of time of the examination. Using only short cases may allow a more efficient examination to be developed.

The combined scores for the cases were comparable across the universities. Such a result suggests that this exam may be useful in a high-stakes assessment. However, further work on reliability of the oral viva examination is required, for example, through the use of generalisability analysis and decision studies. The use of generalisability analysis would allow researchers to establish the variance components within the assessment and indicate how many cases are required to obtain a reliable assessment of CR. Given the focus on the development and validation of the examination in the current paper, future research will directed towards establishing its reliability.

There were some significant differences identified between the scores awarded by one examiner (Examiner 1) compared to the other two examiners. Examiner 1, on average, rated students lower for Question 7. *Please summarise the case so far*; *including your thoughts on differentials*, *examination and treatment strategies* and Question 12. *What would you do if the case was male*/*female*, *older*/*younger*, *more acute*/*chronic*? This result could be explained either by differences in the CR ability of the cohort being examined compared to the other two cohorts, or the fact that Examiner 1 marked these two questions more stringently. Establishing variance components using multiple examiners will provide a greater degree of detail about the influence of the examiner on the assessment itself and will be the subject of future research.

Year 4 and 5 students were recruited for the study. At all three participating institutions these students were enrolled in pre-registration Masters-level programs. The year 5 students were about to graduate from their respective programs at the time of the examination. The finding that year 5 students scored higher in a number of questions and the total score may be related to their higher levels of context specific clinical experience [58]. How CR develops and is utilised in individual practitioners is complex and not necessarily related to length of experience [2, 6]. The development of metacognition, one aspect of CR, appears to differ between general experience and specific contexts of learning [59] and a recent study demonstrated that, although analytic decision-making was found to develop, reflective thinking disposition did not change in students as they progressed through an osteopathic program [58]. Examiners in the current study were not aware of students’ year level before or after the examination so it was not possible to bias this result. Scores appeared to be indicative of the year level of the students. However, it would be valuable to confirm these results in another cohort.

The internal consistency of the assessment rubric was 0.944 and did not improve when items were deleted. As the alpha score can be inflated by substantial relationships between variables (in this case the questions on the rubric), correlation statistics were applied to the questions in order to identify those that may have an impact. Relationships that were very large (>0.70) [48] were identified (Table 3). On review of these large correlations, the authors noted the possibility of overlapping information being obtained from these questions resulting in the high Pearson’s *r* values. Rewording and/or combining these questions will have a two-fold effect: 1) reduce the length of the assessment rubric, making it more efficient for examiners to use; and 2) reduce the alpha score. The assessment rubric used in the present study will be remodeled before use in subsequent studies.

There are some limitations to the present study. Using two cases placed a limit on the breadth of case presentations tested in this exam, although the cases chosen had cues leading to other systems. Further research is planned with a higher number of cases. Small student numbers from each university may not have been representative of the student cohort. There is also a possibility of self-selection bias. Students who chose to participate may have done so for personal reasons like obtaining feedback in preparation for subsequent exams. No one examiner assessed all students in the study, which limited the statistical analyses that could be applied to the data. This was for financial and practical reasons, as one examiner had to fly overseas to conduct the exam. In future examiners from each institution will be trained to perform the assessment to save on cost and potentially improve efficiency.

## Conclusions

The present study has developed an oral examination to assess CR in pre-registration Masters-level osteopathy students. The face and content validity of the exam were established and results suggest that the exam may also be able to differentiate between 4^th^ and 5^th^ year students. These results are promising and further work to establish the reliability of this assessment using generalisability theory is required, along with refinement of the rubric and further examiner training so that it can be implemented as a high-stakes assessment. This research has also contributed to the literature on CR in osteopathy beyond the qualitative descriptions previously published.

## Authors’ information

Paul Orrock is a Senior Lecturer in the osteopathy program at Southern Cross University, Lismore, Australia and an Adjunct Fellow in the College of Health & Biomedicine, Victoria University, Melbourne, Australia. Sandra Grace is a Senior Lecturer in the osteopathy program at Southern Cross University, Lismore, Australia and an Adjunct Research Fellow at the Education for Practice Institute, Charles Sturt University, Bathurst, Australia. Brett Vaughan is a lecturer in the College of Health & Biomedicine, Victoria University, Melbourne, Australia and a Professional Fellow in the School of Health & Human Sciences at Southern Cross University, Lismore, New South Wales, Australia. Rosanne Coutts is the Director of Teaching and Learning for the School of Health & Human Sciences at Southern Cross University, Lismore, Australia.

## Electronic supplementary material

Additional file 1: **Assessment rubric.** (PDF 258 KB)

Additional file 2: **Example ‘short’ and ‘long’ cases.** (DOCX 115 KB)
