# Supplementary material for: Developing a viva exam to assess clinical reasoning in pre-registration osteopathy students
Source: BMC Med Educ. 2014 Sep 19;14:193. doi: 10.1186/1472-6920-14-193 (PMC4179819; doi:10.1186/1472-6920-14-193)
Supplement: Supplementary file 1 — Additional file 1: Assessment rubric. (PDF 258 KB) [file 12909_2014_1023_MOESM1_ESM.pdf]

Osteopathic Clinical Reasoning Viva Assessment

**Attribute/descriptor**	Question(s)	**1**	**2**	**3**	**4**	**5**
**Analysis**<br>Demonstrates interpretation of case information	Q1 How have you interpreted the information given to you so far?	Poor or no attempt to interpret information from case	Limited interpretation of case information	Interprets case information to reveal important patterns, differences & similarities	Thorough interpretation of case information	Comprehensively interprets information
	Q2 Can you integrate this information to give it more meaning?	Poor or no attempt to synthesise relevant information from case	Limited synthesis in use of case information Not able to reveal important patterns, differences and similarities	Synthesises case information to reveal important patterns, differences and similarities	Thorough synthesis of case information to reveal important patterns, differences and similarities	Comprehensively synthesises information. Is able to reveal subtle patterns, differences and similarities
**Heuristics**<br>Makes connections between cues in the case, including the patients context in the additional information	Q1 What are the primary cues in the additional case information?	Unable to identify major cues	Identifies a limited number of cues	Identifies main cues relevant to the case	Identifies majority of cues relevant to the case	Identifies all cues relevant to the case
	Q2 Are there any connections between them?	Unable to make connections	Is able to make limited connection between cues	Connects main cues relevant to the case	Connects the majority of cues relevant to the case	Connects all cues relevant to the case
**Inference**<br>Uses knowledge to generate ideas about differentials, examination and treatment. Discriminates and distinguishes relevant from irrelevant information, recognises inconsistencies, filters information appropriately and identifies gaps in cues collected. Includes consideration of red flags and most likely diagnoses	Q1 Please outline the most important elements of this case and provide some differential diagnoses (DD's)	FAIL<br>Includes irrelevant DD's, omits 'red flags'	FAIL<br>Includes a limited range of relevant DD's<br>Omits major 'red flags'	Discriminates and distinguishes relevant from irrelevant information. Includes major 'red flags' and most likely diagnoses	Includes a range of relevant DD's and 'red flags' using appropriate information	Offers an extensive range of relevant DD's and 'red flags'<br>Recognises and addresses gaps in information
	Q2 Upon what knowledge are you basing your ideas about differentials, examination and treatment?	Poor application of knowledge, with use of irrelevant literature	Limited application of knowledge, with limited use of relevant literature	Appropriate application of knowledge and use of literature	Thorough application of knowledge and appropriate literature	Comprehensive application of knowledge and appropriate literature
**Information processing**<br>Differentials, examination and treatment strategy are organised in a cohesive manner	Q1 Please summarise the case so far; including your thoughts on differentials, examination and treatment strategies	Poor or no attempt at organising and processing information for case diagnosis and management strategies	Limited processing and organisation of information for case diagnosis and management strategies	Information about all aspects of the case are organised in a cohesive manner	Thorough organisation and processing of all aspects of case information	Comprehensively organises and processes information on all aspects of the case. Case information is summarised in a cohesive manner

Attribute/descriptor	Question(s)	1	2	3	4	5
**Logic** Provides sound reasoning for differentials, examination and treatment strategy. Including strategies to rule DDs in and out.	Q1 Give your reasoning for choice of differentials?	Unable to provide sound reasoning for choice of DD's	Limited use of reasoning for choice of DD's	The student provides sound reasoning for choices of DD's	Provides thorough reasoning for choice of DD's	Comprehensive use of advanced reasoning skills for determining DD's
	Q2 What examination and investigation strategies will you use to rule in/out DD's?	No clear strategy for ruling in/out DD's	Limited use of strategy for ruling in/out DD's	Strategies used to rule in/out DD's	Clear logical strategy used to rule in/out DD's	Comprehensive and clearly logical use of strategies
	Q3 Can you now tell me your working diagnosis and the rationale for your treatment strategy?	Illogical working diagnosis and unsound rationale	Limited use of logic in working diagnosis and rationale	Sensible working diagnosis and sound rationale	Thorough and logical working diagnosis and rationale	Comprehensive logic used and rationale given
**Cognition** Thinks 'aloud' about choices in relation to differentials, examination and treatment strategy. Ability to adapt to emerging information after feedback	Q1 Can you tell me about alternative diagnostic or treatment choices if what you have planned doesn't work?	Poor or no attempt to reason alternative options in relation to case	Limited ability to reason aloud alternative options and problem solving strategies in relation to case	Reasons aloud through problem solving strategies in relation to DD's, examination and treatment	Reasons aloud problem solving strategies in relation to all aspects of case	Comprehensively talks through alternatives and problem solving approaches
	Q2 What would you do if the case was male/female, older /younger, more acute/chronic?	Is not able to articulate alternative options	Limited skills in articulating alternative options	Illustrates ability to articulate reasonable alternative options	Thoroughly demonstrates ability to articulate their reasoning and decision(s) in accordance with new information	Comprehensively demonstrates flexibility in reasoning, with ability to adjust DD's and treatment plans according to new information
**Meta- cognition** Demonstrates the ability to reflect on their reasoning process, including with regards to osteopathic principles (body as a unit, structure and function inter-related, self healing capacity)	Q1 What are your thoughts about how your handling of this case could have been improved?	Poor or no attempt at reflection of the strengths and weaknesses in their reasoning process	Limited ability for reflection of the strengths and weaknesses in their reasoning process	Student is able to reflect of the strengths and weaknesses in their reasoning process	Demonstrates thorough reflection of the strengths and weaknesses in their reasoning process	Comprehensively reflects on the strengths and weaknesses in their reasoning process
	Q2 How did the osteopathic principles influence your reasoning in this case?	Poor or no attempt at reflection on how osteopathic principles have influenced their reasoning process	Limited ability for reflection on how osteopathic principles have influenced their reasoning process	Student is able to reflect on how osteopathic principles have influenced their reasoning process	Demonstrates thorough reflection on how osteopathic principles have influenced their reasoning process	Comprehensively reflects on how osteopathic principles have influenced their reasoning process
**Overall performance**						

Please comment on issues in the students performance, especially if they have failed a section or the whole case

Comments…
