# Supplementary material for: Developing a viva exam to assess clinical reasoning in pre-registration osteopathy students
Source: BMC Med Educ. 2014 Sep 19;14:193. doi: 10.1186/1472-6920-14-193 (PMC4179819; doi:10.1186/1472-6920-14-193)
Supplement: Supplementary file 2 — Additional file 2: Example ‘short’ and ‘long’ cases. (DOCX 115 KB) [file 12909_2014_1023_MOESM2_ESM.docx]

| **Case 1 - short** |
| --- |
| **1. Presenting complaint**  35-yr old female presents with acute low back pain referring into left buttock following a yoga class |
| **Medical and diagnostic thoughts – further information**  Student answers |
| **2. Examiner gives extra information**  Doing yoga teacher training, had first child 9 months ago, vegetarian, was a lawyer in the city for 10 years “burnt out”, reports spotting (blood) between periods lately. |
| **DD**  Student answers |
| **3. Physical examination/Investigation findings**   - Kemps positive for left familiar buttock pain - Slump negative - Plain lumbosacral x ray NAD - Negative SLR |
| **Primary diagnosis**  Student answers  **Flags/features:**  Student answers  **Management Plan**  Student answers |
| **Case 12 – long** |
| **1. Presenting complaint**  60 year old male executive who spends the majority of his work week flying back and forth between their Melbourne and Sydney offices. He has two adult children and one grandchild. He is quite overweight and is visibly out of breath as he sits down in your treatment room.  Recent onset of left shoulder pain, diffuse in nature and hard to pinpoint. It started after a recent game of squash. There was no particular incident during the game, but he was quite red and out of breath after only 5 minutes and thinks he may have ‘tweaked it’ while being exhausted and not concentrating on his game. Barry confirms that he is indeed left handed. The pain does seem to come and go and he hasn’t really tried to link it to any particular movement or action. He has tried taking a few Nurofen, which haven’t helped and Panadeine only gave some temporary relief. |
| **Medical and diagnostic thoughts – further information**  Student gives answers |
| **2. Examiner gives extra information**  10 years ago he had injured this shoulder quite badly and had given up playing squash as a consequence. He had sought physio treatment at the time, and thinks that the diagnosis was a small tear through the ‘supra spine’ muscle, but he cannot really remember. It had never fully recovered, and he admits that the shoulder can be very stiff, particularly during winter, and he has trouble reaching over his head on aircraft to get his bags.  he likes his food a little too much, no regular exercise  hasn’t been to the GP in the last 8 years as he is very rarely sick  quite often out of breath. occasional bouts of ‘heartburn’ after big meals but has not had this checked by anyone and he finds that chewing a few heartburn lollies helps.father had died of a heart attack at 73, after having high cholesterol and blood pressure for ‘quite a few years’.  His diet consists mainly of rich restaurant food as he is constantly interstate on business. He very rarely cooks at home, and on the weekends he usually takes his wife out for take away and a glass of wine now that their children are all grown up and moved out. |
| **DD**  Student gives answers |
| **3. Physical examination/Investigation findings**  Cardio:   - Peripheral pulses: present bilaterally - Heart Rate/rhythm: 96 bpm - Jugular Venous Pulsations: normal - Carotid Pulsations: normal - Apical Beat: strong and normal - Abnormal heart sounds/murmurs: none - BP 150/98   Special Tests:   - Empty Can positive L - Speeds positive L - AC provocation negative - Apprehension/Relocation negative - Cervical Quadrant -ve   Further Examinations:   - Ultrasound L shoulder: evidence of calcific supraspinatus tendon, and a small 3mm tear in the musculotendinous juntion. |
| **Flags/features:**  Student answers |
| **Management Plan**  Student answers |
